# A New Sutureless Illuminated Macular Buckle Designed for Myopic Macular Hole Retinal Detachment

**DOI:** 10.1155/2017/6742164

**Published:** 2017-03-16

**Authors:** Ahmed M. Bedda, Ahmed M. Abdel Hadi, Mohamed Lolah, Muhammad S. Abd Al Shafy

**Affiliations:** ^1^Ophthalmology Department, Faculty of Medicine, Alexandria University, Alexandria 21526, Egypt; ^2^General Ophthalmology Hospital of Alexandria, Alexandria 21547, Egypt

## Abstract

*Purpose*. To report the anatomic and visual results of a new sutureless illuminated macular buckle designed for patients with macular hole retinal detachment related to high myopia (MMHRD). *Design*. Prospective nonrandomized comparative interventional trial. *Methods*. Twenty myopic eyes of 20 patients (mean age, 51.4 years; range, 35–65 years) presenting with MMHRD with a posterior staphyloma, in whom the new buckle was used, were evaluated. The buckle used was assembled from a 5 mm wide sponge and a 7 mm wide silicone tire; it was fixed utilizing the sterile topical adhesive Histoacryl Blue (B Braun, TS1050044FP) which polymerizes in seconds upon being exposed to water-containing substances. The primary outcomes measured included aided visual acuity (BCVA) and optical coherence tomography (OCT) findings. The mean follow-up period was 6 months. *Results*. Postoperatively, the MH closure was identified by OCT in 8 (40%) eyes. The mean BCVA increased from 0.11 to 0.21 (*p* < 0.005). The axial length of the eyes included decreased from 30.5 mm preoperatively to 29.8 mm (*p* = 0.002) postoperatively. *Conclusion*. Preparation of the new sutureless macular buckle is simple and easy. Illumination of the terminal part of the buckle ensures proper placement. Histoacryl Blue is effective in fixing the buckle in its place for at least 6 months with no reported intra- or postoperative complications.

## 1. Introduction

Progressive myopia is a relatively frequent condition affecting all ocular structures, including the vitreous, the retina, the choroid, and the sclera. Globe elongation with subsequent development of posterior staphyloma represents the hallmark of the disease and can be complicated by myopic foveoschisis and myopic macular hole with secondary retinal detachment [1].

Other factors implicated in the pathogenesis are anteroposterior traction caused by the vitreous cortex, tangential forces due to the epiretinal membranes (ERMs) or the internal limiting membrane (ILM), and the stretched retinal arteries [2].

With the revival of macular buckling as a noninvasive surgical solution for these cases, several published reports describe a success rate comparable to or even higher than that of pars plana vitrectomy which was considered the preferred surgical procedure for this relatively complicated type of detachment [3, 4].

Difficulties with EMB prevented its establishment as the gold standard treatment for myopic macular hole detachment; such difficulties include accurate placement under the macular hole, with a sufficient indentation height to alleviate the stretched macular area [5].

Fibre-optic-guided Ando plombe was used few years back [5], which improved the success rate of such surgery.

Despite the 100% and 40% rates of retinal reattachment and MH closure, respectively, using the fibre-optic-guided Ando plombe, scleral perforation occurred in 15% of the cases, which was significantly higher than that of the vitrectomy group operated by the same surgeon in a recent study published during 2015 [4].

Consequently, to avoid any damage during buckle fixation and to enhance visualization, we propose the use of a new sutureless fibre-optic-guided macular buckle, which allows better visualization of the indenting heel, as well as placing it correctly under the centre of the fovea.

## 2. Patients

Recruitment of cases took place between February 2015 and August 2015. MH was defined as the presence of a foveal full thickness based on the fundus examination. Twenty myopic eyes from 20 patients with a high degree of myopic error, defined as eyes with axial length of >30 mm or greater (measured by the calliper during a B-scan US), with macular hole retinal detachment were selected to be included in the study. Additional inclusion criteria were the absence of any stage of proliferative vitreoretinopathy; no history of posterior segment eye surgery; intraocular pressure lower than 20 mmHg; absence of any systemic disease that might confound the visual function, for example, diabetes; and absence of history of ocular trauma.

## 3. Methods

This was a prospective, interventional case series conducted at a tertiary referral centre in Alexandria, Egypt. The study was conducted in accordance with the Declaration of Helsinki and its subsequent amendments. This research protocol and its amendments were approved by the Ophthalmology Department of the Alexandria University Institutional Review Boards and Ethics Committees. Explanation about the procedure and its duration was given to the subject of the research in clear, understandable words. Each patient was informed about the liable reasonable risk. All patients provided written informed consent. Confidentiality was assured.

In all cases, the following examinations were performed preoperatively.

Best-corrected visual acuity (BCVA) was assessed by using Snellen testing. Applanation tonometry was carried out. Dilated indirect binocular ophthalmoscopy with scleral depression was performed to exclude the presence of peripheral tear. Slit lamp examination supplemented with a plus 90-D lens to confirm the diagnosis was done. Fundus photograph was taken; axial length was measured utilizing the A/B scan vector method to avoid fallacies in the myopic eyes with detached retinas by means of a 10 MHz probe (Echoscan US 4000, Nidek Inc., Fremont, CA); and optical coherence tomography (OCT; Cirrus HD-OCT 4000, version 5.0, Carl Zeiss Meditec) before operation was performed whenever feasible.

In certain cases, OCT was not useful or even possible to perform either due to vitreous opacities obscuring the view of the macula or due to a bullous central retinal detachment with no useful data obtained as the area which might contain the hole was out of range of the machine used. Postoperatively at 1 day, 15 days, and 1, 3, and 6 months, patients were examined. Slit lamp examination was done; fundus examination was attempted in all patients on day 1 to assess the condition of the retina, and thereafter during all postoperative visits. Fundus photography and OCT were ordered when needed.

## 4. Surgical Technique

The surgical technique was used in 20 patients with high myopia and central retinal detachment who were referred to an Alexandria vitreoretinal centre in Alexandria, Egypt (See Supplemental Video available online at https://doi.org/10.1155/2017/6742164). All operations were performed by the same surgeon (AMB).

**Figure 1:**
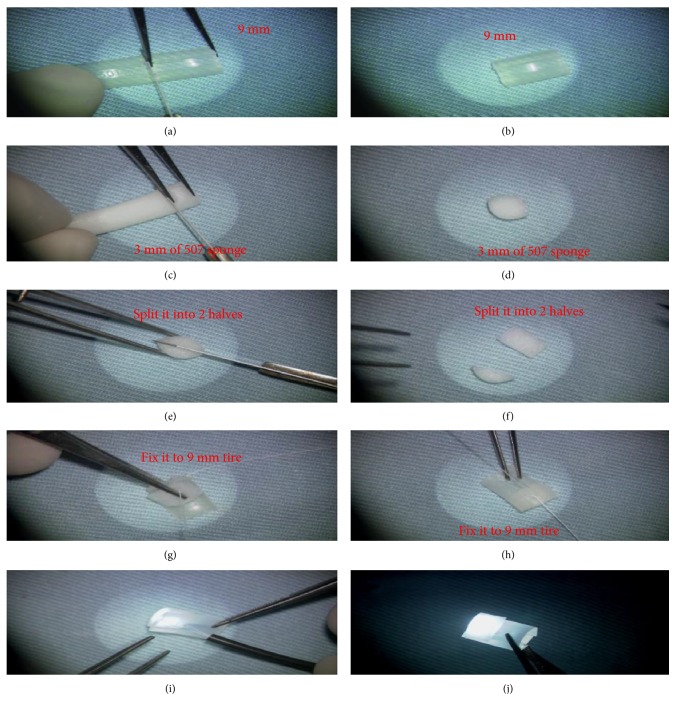
(a–f) Macular buckle assembled from a 5 mm wide sponge and a 7 mm wide silicone tire. A 9 mm segment from the tire was cut; a 6 mm segment from the sponge was split into two halves. (g, h) Both were sutured together so that the convex part is made facing the sclera. (i, j) Before starting the operation, a track is prepared for a fibre-optic light in which a disposable 25 g chandelier was inserted in the indenting head.

The buckle used was assembled from a sponge (width, 5 mm) and a silicone tire (width, 7 mm) readily available in the vitreoretinal operating room. A 9 mm segment from the tire was cut; a 6 mm segment from the sponge was split into two halves and sutured to the tire so that the convex part is made facing the sclera (Figure 1).

Before starting the operation, a track is prepared for a fiber-optic light. A disposable chandelier fibre (25 gauge/0.5 mm) (Geuder AG, Heidelberg, Germany) was inserted in the indenting head (Figure 1).

The surgical steps included performing a superotemporal conjunctival peritomy, careful exposure of the superior and inferior oblique muscles so as to identify the posterior edge of the inferior oblique insertion marking the exact position of the transverse long posterior ciliary artery (TLPCA).

Subretinal fluid evacuation was attempted in cases where RD was so extensive to reach the peripheral retina. In those cases, with shallow retinal detachment limited to the posterior pole, a paracentesis was done instead as posterior drainage was too risky to perform and very difficult without muscle disinsertion. This was done to allow for adequate indentation of the tire at the macular area.

**Figure 2:**
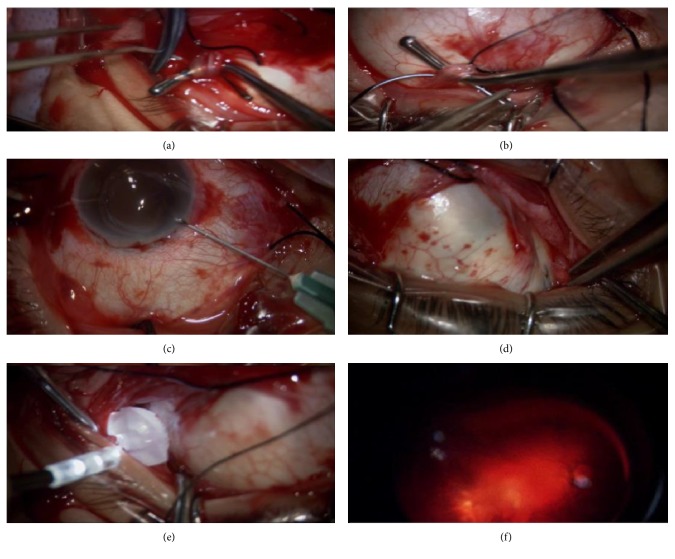
Surgical steps: (a) exposure and hanging of the inferior oblique; (b) exposure and hanging of the superior oblique; (c) paracentesis or evacuation of subretinal fluid was attempted; (d) identification of the transverse long posterior ciliary artery; (e) temporary placement of the macular buckle along the vessel course; (f) confirmation of the position of the buckle head by the aid of binocular indirect ophthalmomicroscopy.

Preliminary place the buckle along the prementioned vessel course. Turn on the fiber-optic light previuosly fixed to the buckle when the buckle head was thought to be accurately placed. With the aid of the binocular indirect ophthalmomicroscopy (Oculus BIOM5, OCULUS Optikgeräte GmbH) system (BIOM), the glowing head position could be seen easily (Figure 2).

Now, the macular buckle was fixed in place by utilizing the sterile topical adhesive Histoacryl Blue (B Braun TS1050044FP). This is a sterile liquid topical adhesive composed of n-butyl-2-cyanoacrylate monomer. Histoacryl Blue—supplied in 0.5 ml single-use ampoules—is colored with the dye D&C Violet #2 in order to easily see the thickness of its applied layer. The tissue adhesive polymerizes in seconds upon being exposed to water or water-containing substances like a human tissue. The buckle's head could still be adjusted and positioned under the fovea before the tissue adhesive fixes it in place.

At the completion of the surgery, filtered air was injected in cases where subretinal fluid drainage was done to restore normal IOP. Sutureless macular buckling with fibre-optic-guided episcleral buckle insertion was the only procedure performed in all patients.

## 5. Results

The study included 20 highly myopic eyes from 20 patients attending a specialized vitreoretinal centre in Alexandria, Egypt. Demographic data and the axial lengths, before and after surgery of the eyes as measured by B-scan US, are shown in Table 1.

**Figure 3:**
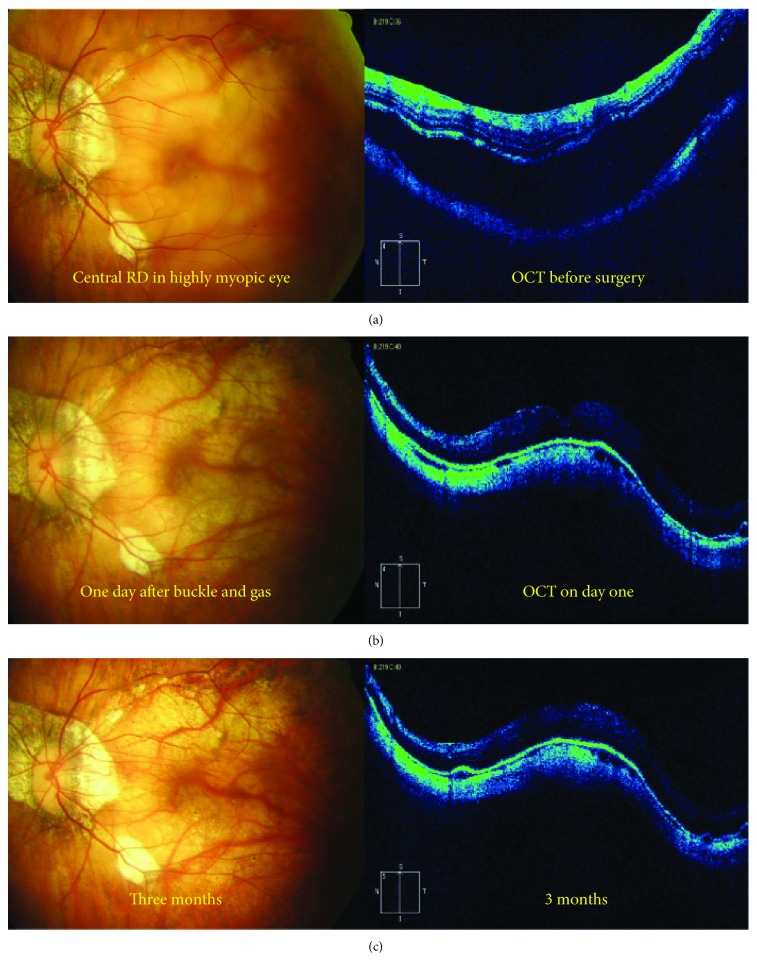
(a) Fundus photograph of a highly myopic eye with central macular detachment; the corresponding preoperative OCT was unable to detect the macular hole, in spite of being detected by high-magnification contact biomicroscopy, probably due to vitreous opacities preventing a detailed OCT examination of the area harboring the hole. (b) Only one day after the buckle, the fluid is gone and the retina is attached; this was confirmed by OCT. (c) Three months later, the fundus photograph shows the retina completely in place with no recurrence of detachment; also OCT shows attachment of the macula with a convex configuration of the posterior pole.

**Figure 4:**
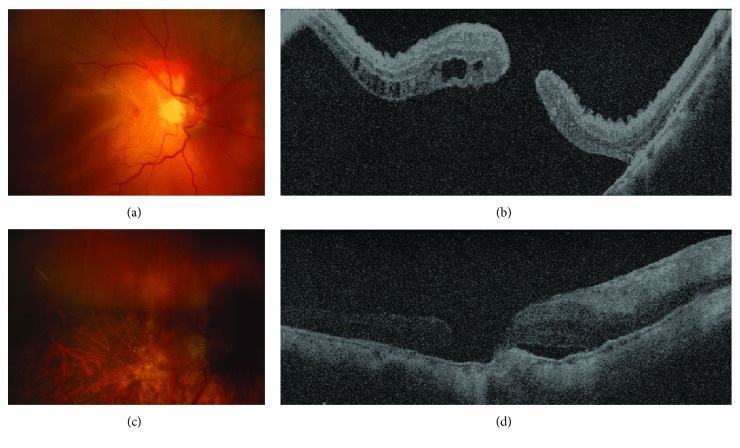
(a) Fundus photograph showing an eye with macular hole retinal detachment, confirmed by OCT (b). (c) Only 3 days after surgery, the retina was totally attached, which was confirmed by OCT (d).

The study sample included 20 eyes from 20 high myopia patients (mean age, 51.4 years; range, 35–65 years). Nine were males (45%) with a mean age of 48 years, while 11 were females with a mean age of 54.2 years. Fundus photography showed retinal reattachment in all eyes. OCT showed a convex configuration of the posterior pole with foveal reattachment in all eyes (Figures 3 and 4).

There was a statistically significant difference in axial length before (30.5 mm) and after (29.8 mm) the procedure in the studied eyes, *p* = 0.002. Likewise, the BCVA after the surgery (0.21) was statistically better than the BCVA before the surgery (0.11), *p* < 0.005.

At the end of the six-month follow-up period, normal foveal contour and architecture were evident on OCT and macular hole closure occurred in 8 (40%) cases.

The mean BCVA of the eyes where the holes have closed was 0.28 versus a mean BCVA of 0.16 in the eyes with persistent opened holes.

## 6. Discussion

The presence of a marked posterior staphyloma in high myopia patients clearly affects the surgical outcomes of MHRD surgeries. This observation paved the way for the resurrection of the macular buckling, which primarily addresses the posterior scleral elongation caused by the staphyloma.

Nowadays, OCT allows detailed imaging of myopic foveoschisis, myopic macular hole, and even early retinal detachment, a diagnosis difficult to be given for sure by fundus examination [6, 7]. The prevalence of foveoschisis in highly myopic eyes ranges from 9% to 34% depending on the series [8–10].

The use of macular buckling in MHRD was proven to have many advantages over vitrectomy like avoiding cataract progression and iatrogenic breaks, which are common risks of vitrectomy [11].

In the present study, the macular buckle used was prepared from materials readily available in the vitreoretinal operation room. This buckle has the same advantages as the Ando plombe (production discontinued) of the safe placement on the sclera without extraocular muscle disinsertion, reducing the potential damage of the nerves and vessels in the posterior pole. It has a much shorter length of 9 mm in comparison to the 21–29 mm length range of the Ando plombe. Moreover, accurate placement over the region of the fovea containing the hole externally was guaranteed by the use of fibre-optic light [4].

The development of a sutureless buckle was the natural next step in the evolution of the fibre-optic-guided macular buckle because of the high rate (15%) of scleral perforation in the macular buckle group in the original series performed by the authors [4].

In the present study, foveal reattachment was achieved in all patients as confirmed by fundus examination postoperatively and optical coherence tomography. Moreover, all patients had an improvement in their BCVA, which was statistically significant (*p* < 0.005). The axial length decreased from 30.5 mm preoperatively to 29.8 mm postoperatively, which was again statistically significant. This was due to the indenting effect of the buckle. In all cases, the macular buckle was easily positioned without the need for subsequent procedures such as repositioning or replacement. Extraocular muscle cutting which was a mandatory step in the macular buckle developed by Siam et al. was not needed [12]. The surgical technique described in their study involved cutting the superior oblique as well as placing two posterior sutures as close as possible to the optic nerve without causing damage to the posterior ciliary vessels.

There were no intraoperative or postoperative complications in any of the 20 eyes operated; consequently, the new proposed tissue adhesive was successful in fixing the buckle in its place with no reported migration of any of the buckle still at the end of the follow-up period.

The macular hole closure was identified by OCT in 8 (40%) eyes. This was similar to the macular hole closure rate in the original series, where it was suggested that even applying the macular Ando plombe alone was probably enough to counteract the anteroposterior traction exerted by the staphyloma. By changing the posterior eyewall from a concave into a convex shape, the retina tends to reattach to the underlying RPE, thus also facilitating the MH closure in some cases [4].

In spite of the aforementioned advantages of the sutureless macular buckles, several studies reported the effectiveness of a primary combined surgery which includes both PPV and the episcleral approach. In the series by Alkabes et al. [13], postoperative results in previously untreated MHRD cases (group 1, 21 eyes) were compared to those obtained in recurrent cases (group 2, 21 eyes). Final retinal reattachment and MH closure rates in previously untreated MHRDs were 100% and 81%, respectively. However, the same rates were slightly lower in case of recurrent MHRDs, reaching 90.5% and 57%, respectively. Based on these results, the authors suggested that PPV combined with MB might be considered as the first surgical approach especially in naïve MHRD cases.

The eyes where the hole closed achieved a mean BCVA of 0.28 versus a mean BCVA of 0.16 in the eyes with persistent opened hole; this was statistically significant (*p* < 0.005). This finding was highlighted in many other studies [11, 13].

## 7. Conclusion

Preparation of the new sutureless macular buckle is simple and easy. Moreover, illumination helps to ensure proper placement. The tissue adhesive (Histoacryl Blue) is effective in fixing the buckle in its place for at least 6 months with no reported intra- or postoperative complications in the 20 eyes studied.

## Supplementary Material

The Surgical steps include exposure and hanging of the inferior oblique and superior oblique muscles. Paracentesis or evacuation of subretinal fluid is attempted to soften the eye; Identification of the transverse long posterior ciliary artery which marks the position of the fovea internally, temporary placement of the macular buckle along the vessel course is done. Confirme the position of the buckle head by the aid of binocular indirect ophthalmomicroscopy. The tissue adhesive- blue colored - is put before closure of the conjunctiva to conclude the surgery.

## Figures and Tables

**Table 1:** Demographic data, axial length, BCVA, and macular hole closure rate.

Number	Sex		Axial length (mm), *p* = 0.002^∗^		BCVA (decimal), *p* < 0.005^∗^		Macular hole closure
	Male	Female	Preop	Postop	Preop	Postop	
20	9 (45%)	11 (55%)	30.55	29.8	0.11	0.21	8 eyes closed (40%)

^∗^Statistically significant at *p* ≤ 0.05.
